# Supplementary material for: Content-rich biological network constructed by mining PubMed abstracts
Source: BMC Bioinformatics. 2004 Oct 8;5:147. doi: 10.1186/1471-2105-5-147 (PMC528731; doi:10.1186/1471-2105-5-147)
Supplement: Additional File 2 — The original results of the above study (non-essential files are deleted to keep the file size under the limit set by BMC bioinformatics). [file 1471-2105-5-147-S2.bz2 › chilibotAdditionalFile2/dip20/186ID8627325E631/html/ADA_ADORA1.html]

 


 **ADA** and **ADORA1** 
  
Found 67 abstracts in PubMed, retrieved 20.  
 

 What does Google say? 
 PDF only 
| .edu only 

---

**Interactive relationship** (e.g. stimulation, inhibition, etc)

**Inhibitory relationship**- The KCl induced release of 3H ACh, but not the release of 14C GABA, was potentiated when adenosine A1 receptor  [ **ADORA1** ]  activation was prevented by perfusing the cells with adenosine deaminase  [ **ADA** ]   **ADA**  or with dipropyl 8 cycloentylxanthine DPCPX.  Ref: 10661490 Brain Res, 2000
- Using these cultures we observed that both 3H ACh release, which was largely Ca2 dependent, and 45Ca2 influx, evoked by depolarization with 50 mM KCl, were increased when adenosine A1 receptor  [ **ADORA1** ]  activation was prevented by removal of endogenous adenosine with adenosine deaminase  [ **ADA** ] , or by application of the A1 receptor antagonist DPCPX.  Ref: 9858381 Neuroreport, 1998
**Stimulatory relationship**- **ADA**  5 U ml stimulated the release of both hormones P< ; 0.01 as also did the selective adenosine A1 receptor  [ **ADORA1** ]  antagonist, dipropyl 8 cyclopentylxanthine DPCPX, 0.1 &.  Ref: 9930581 Regul Pept, 1999
**Neutral relationship**- This  **ADA**  VII 5 U 30 microgram effect could be mimicked by the selective adenosine A1 receptor  [ **ADORA1** ]  antagonist dipropyl 8 cyclopentylxanthine DPCPX, 10 6 mol l.  Ref: 9843917 Am J Physiol, 1998

**Non-interactive relationship** (e.g. studied together, co-existance, homology, etc.)

- this effect was blocked by the adenosine A1 receptor  [ **ADORA1** ]  antagonist cyclopentyltheophylline but was not affected by adenosine deaminase  [ **ADA** ] .  Ref: 9690876 Br J Pharmacol, 1998
- The post ATP facilitation could be prevented by the adenosine A1 receptor  [ **ADORA1** ]  antagonists 8 phenyltheophylline or dipropyl 8 cyclopentyltheophylline 50 nM, or superfusion of adenosine deaminase  [ **ADA** ] .  Ref: 11104829 Eur J Pharmacol, 2000
- The release of 3H ACh evoked by 50 mM KCl was mostly Ca2 dependent, and it was increased in the presence of adenosine deaminase  [ **ADA** ]  and in the presence of dipropyl 8 cyclopentylxanthine DPCPX, an adenosine A1 receptor  [ **ADORA1** ]  antagonist.  Ref: 9721733 J Neurochem, 1998
- When extracellular adenosine was inactivated by adenosine deaminase  [ **ADA** ] , phenylisopropyladenosine, an adenosine A1 receptor  [ **ADORA1** ]  agonist, potentiated the insulin induced accumulation of PtdIns, 5 P3.  Ref: 10391887 J Biol Chem, 1999
